# SARS-COV-2 IgG specific antibodies persistence in recovered COVID-19 individuals and its association with severity and time of illness

**DOI:** 10.1016/j.nmni.2023.101096

**Published:** 2023-02-03

**Authors:** Abdol Sattar Pagheh, Arash Ziaee, Khadijeh Abrari Romenjan, Fatemeh Rezaei, Babak Bahman, Effat Alamzadeh, Samira Elhamirad, Masood Ziaee

**Affiliations:** a Infectious Diseases Research Center, Birjand University of Medical Sciences, Birjand, Iran; b Department of Neuroscience, Mashhad University of Medical Sciences, Mashhad, Iran; c Islamic Azad University of Chalous Branch, Chalous, Iran

**Keywords:** Coronavirus nucleocapsid protein, COVID-19, IgG, SARS-CoV-2, Severity

## Abstract

In order to accurately interpret the immune response to COVID-19, it is critical to know how long serum antibodies to COVID-2 persist. This study aimed to describe the serum IgG responses to SARS-CoV-2 in patients with mild, moderate, and severe COVID-19 infection in Birjand, South Khorasan province, Iran. The study was performed on individuals whose COVID-19 disease was confirmed by RT-PCR and recovered from the disease. After completing the questionnaire, blood samples were collected from 4 different groups based on the time of the test at two, four, six, and eight months' post-recovery. Then, SARS-COV-2 virus-specific IgG nucleocapsid antibody level in patients was measured using the enzyme-linked immunosorbent assay (ELISA). In total, 206 patients (mean age 44.19 ± 14.9, 51% man) were included in the survey. Serum prevalence of specific IgG antibodies in patients with mild, moderate, and severe COVID-19 disease was 51.5%, 64% and 78.9%, respectively. Furthermore, serum prevalence of COVID-19 specific IgG antibody level in two, four, six, and eight months after recovery were 80.8, 69.1, 43.2 and 41.8%, respectivel**y** (p < 0.05). The multiple logistic regression model showed that the variables of age and the time elapsed after recovery had a significant relationship with the positive antibody test of recovered COVID-19 patients (P < 0.05). But other variables had no significant relationship with the result of antibody test (P > 0.05). In the present report, we attempted to characterize the antibody response against SARS-CoV-2 in patients with mild, moderate, and severe COVID-19, with the aim of better elucidating the humoral immune response after recovery from SARS-CoV-2 infection.

## 1 Introduction

On December 29, 2019, physicians of a hospital located in Wuhan, China, noticed unusual cases of patients with pneumonia. Unusual pneumonia cases were reported to the World Health Organization on December 10. Thus, the SARS-CoV-2 virus was detected, which caused the COVID-19 pandemic. This virus has infected all countries and has taken millions of people's lives [1]. Symptoms of this disease range from mild fever, headache, myalgia, and rarely gastrointestinal disorders to severe lung damage and death. The virus can be transmitted through the respiratory tract [2,3]. The novel coronavirus can have irreversible impacts on society, especially for at-risk patients such as cardiovascular disease patients, diabetes patients, people with compromised immune systems, and the elderly. On the other hand, for the health care system of a country, there are consequences like astronomical costs such as performing diagnostic interventions, treatment, and long-term hospitalization for a considerable number of patients, the loss of human resources in a society, and the prevalence of psychological problems [4,5]. In the current unprecedented situation, the number of cases is increasing rapidly.

It should be acknowledged that it is crucial to find the suspected cases as soon as possible and to isolate them quickly to stop the spreading of the infection. Until now, the conventional and globally accepted diagnostic test method is RT-PCR. New coronavirus nucleic acids can be detected in samples such as nasopharyngeal swabs and lower respiratory tract secretion [6,7]. At the same time, serological testing methods for detecting COVID-19 antiviral antibodies have been developed and used. Diagnosing the acute phase of the disease, asymptomatic carriers, and recovered individuals is possible by determining specific antibodies to the SARS-CoV-2 virus by serological testing such as ELISA.When the body is exposed to a virus; it takes time for the immune response to produce antibodies. The amount of antibodies varies for each individual and increasing the level of the specific antibodies can mount a stout defense against the virus. Preliminary studies show that patients with the novel Coronavirus who have survived the disease naturally have a significant amount of specific antibodies in their bodies. Serological testing is the principal means for detecting the presence of a specific antibody reaction and the extent to which the virus has spread among the population [[7], [8], [9]].

Seroprevalence studies of disease-specific antibodies can provide valuable data on recent or past infection cases. Serum monitoring of infection in people with a history of COVID-19 helps assess the extent of an effective immune response against this disease [[8], [9], [10]].So far, there is evidence for immune responses against SARS-CoV-2, but the timing of seroconversion and the resulting increase in antibodies is not well understood. Notably, the association between positive serum antibody levels and protection against re-infection and the duration of protective immunity is unclear [[9], [10], [11]]. The presence or absence of protective immunity due to infection or vaccination will affect the transmission and severity of the disease [12,13]. Given these factors, the study aimed to evaluate the persistence of IgG against SARS-COV-2 among recovered COVID-19 individuals based on the time elapsed since recovery and its association with sex, age, clinical symptoms, and disease severity.

## 2 Methods

### 2.1 Ethical considerations

The present study was conducted the period February 2021-February 2022 in Birjand, South Khorasan, Iran. Before the study and sample collection, the Ethics Committee of the Birjand University of Medical Sciences (IR.BUMS.REC.1399.104) approved the research protocol; andinformed consent was obtained from all the participants.

### 2.2 Study design

The inclusion criteria for this study were individuals with a positive real-time reverse transcription polymerase chain reaction (RT-PCR) for SARS-CoV-2 patients who had recovered. Exclusion criteria were the subjects with a history of COVID-19 vaccination and those without a definitive real-time RT-PCR result, as well as individuals who were re-infected with covid-19 during the laboratory examination period. The outcome of this study was measuring SARS-CoV-2-specific IgG antibodies in two, four, six, and eight months' post-recovery. The questionnaire consisted of information about demographic characteristics (age, sex, education, occupation, and underlying disease), hospitalization, clinical symptoms, the time elapsed since recovery, length of hospital stay, and disease severity. The classification of COVID-19 disease severity was according to the clinical management of COVID-19 guidance in three groups: mild, moderate and severe. Hereby, mild illness included symptomatic patients who met the case definition for COVID-19 without evidence of viral pneumonia or hypoxia. Moderate illness in adolescent or adult included clinical signs of pneumonia (fever, dyspnoea, cough, fast breathing) but no signs of severe pneumonia, including oxygen saturation or SpO2 ≥ 90% on room air. Patients with mild and moderate illness may not require emergency interventions or hospitalization; however, isolation is necessary for all suspect or confirmed cases to contain virus transmission. It should also be noted that adolescent or adult with clinical signs of pneumonia and the following criteria were diagnosed with severe COVID-19: SpO2< 90% on room air and respiratory rate >30 breaths/min [14]. Based on time post-recovery, the patients were categorized into four groups included two, four, six, and eight months after recovery from SARS-CoV-2 infection. After obtaining written, informed consent, blood samples were initially collected from all participants. Serum isolation was performed by centrifugation at 5000 g for 5 minutes. The serum samples were kept at -20 °^C^ for serological tests.

### 2.3 Enzyme-linked immunosorbent assay (ELISA)

Serological detection of anti-N (nucleocapsid) SARS-CoV-2 antibodies was performed using SARS-CoV-2 IgG ELISA kits (PishtazTeb Company, Tehran, Iran) according to the kit manufacturer's instruction. Briefly, 100 μL of diluted samples (1:101) was applied to a 96-well microplate (coated with N protein) and incubated at 37°C for 30 minutes. The plate was washed five times and shaken. Then, 100 μl of anti-human IgG-HRP conjugate was added to each well ‏and incubated at 37°C for 30 minutes. After secondary washing, 100 μL of the chromogen-substrate solution was added to the wells and incubated at 37°C for 15 minutes. Finally, the enzyme-substrate reaction was stopped by adding 100 μL of the stop solution to the wells. Each well's optical density (OD) was measured using a microtiter plate reader at 450 nm (630 nm filter as the reference filter). The antibody concentration was calculated as the ratio of OD to the cut-off value. Accordingly, the samples higher than 1.1 considered as positive, and those less than 0.9 assigns as negative, and those be-tween these two values, 0.9-1.1, were considered as suspicious or equivocal, and tested again with fresh serum sample after a while.

### 2.4 Statistical analysis

Statistical analyses were performed with SPSS software v20.0 (IBM Corp. Released 2011. IBM SPSS Statistics for Windows, Version 20.0. Armonk, NY: IBM Corp.). A P-value less than 0.05 was considered significant. Quantitative and qualitative variables were presented as mean (standard deviation [SD]) and frequency (%), respectively. Mann-Whitney and Kruskal-Wallis tests were used to compare IgG levels by demographic variables and underlying disease records. Chi-square and extended Fisher (Fisher-Freeman-Halton) were performed to measure the categorical variables. Furthermore, a multiple logistic regression model was utilized to determine the factors associated with positive specific IgG antibodies.

## 3 Result

### 3.1 Characteristics of the recovered COVID-19 subjects

The study was conducted on 206 COVID-19 subjects in four different time frames post recovery including 2 months (n = 52), 4 months (n = 55), 6 months (n = 44), and 8 months (n = 55) in different groups with mild (n = 101), moderate (n = 86), and severe (n = 19) clinical symptoms. Of all participants in the three groups, 51% (n = 105) were male, and 9.7% (n = 20) had a history of underlying diseases. The mean age of patients who recovered from COVID-19 with positive and negative IgG results was 47.95 ± 14.82 years and 38.71 ± 11.19 years, respectively.

### 3.2 Association between Specific-IgG with demographic and clinical characteristics of the patients

Out of 206 patients, 122 (59.2%) were positive for the presence of COVID-19-specific IgG antibodies during the periods divided into 4 time periods. Among the positive cases, 62 (50.8%) were female, and no significant relationship was observed between gender and specific antibodies against COVID-19 (*P* = 0.53). The results showed a significant relationship between COVID-19-specific antibodies with the variables of hospitalization (*P* = 0.006), level of education (*P* = 0.046), and underlying disease of the studied subjects (p < 0.01) and the frequency distribution of COVID-19 disease symptoms by IgG test result can be seen (Table 1). Furthermore, based on the independent t-test, there was a statistically significant difference between the mean age of patients with IgG results (p < 0.001). The age of the severe group was higher than the other two groups (*P* < 0.001). Table 2 shows the frequency distribution of COVID-19 disease symptoms based on all positive IgG cases.

**Table 1:** The relationship between IgG test result and demographic and clinical characteristics of the patients improved after Covid-19

Variables	Total	IgG		P
		Negative Positive		
**Age**				
Standard deviation ± mean	44.1 ± 14.18	38.71 ± 11.19	47.95 ± 14.82	0.001∗
30 ≥ yrs	33 (100)	21 (63.6)	12 (36.4)	0.001∗
31-45	85 (100)	41 (48.2)	44 (51.8)	
60- 46	66 (100)	20 (30.3)	46 (69.7)	
≥60	22 (100)	2 (9.1)	20 (90.9)	
**Sex**				
Female	101 (100)	39 (38.6)	62 (61.4)	0.536
Male	105 (100)	45 (42.9)	60 (57.1)	
**Hospitalization**				
No	106 (100)	53 (50)	53(50)	0.006∗
Yes	100 (100)	31 (31)	69 (69)	
**Education**				
Lower than Diploma	31 (100)	6 (19.3)	25 (80.7)	
Diploma	34 (100)	14 (41.2)	20 (58.8)	0.046
Bachelor's degree	89 (100)	43 (48.3)	46 (51.7)	
Higher than bachelor's degree	49 (100)	21 (42.9)	28 (57.1)	
**Occupation**				
Housekeeper	33 (100)	9 (27.3)	24 (72.7)	
Retired∖employee	134 (100)	57 (42.5)	77 (57.5)	0.20
Other	39 (100)	18 (46.2)	21 (53.8)	
**Underlying disease**				
Negative	186 (100)	81 (43.5)	105 (56.5)	0.01∗
Positive	20 (100)	3 [15]	17 (85)	

#Values are reported as a number (percentage) or standard deviation ± average. ∗ Significant at the 0.05 level.

**Table 2:** Comparison of the frequency distribution of COVID-19 disease symptoms in patients with negative and positive IgG test results

Variables	Total	IgG		P-value
		Negative Positive		
**Fever**				
Negative	101 (100)	46 (45.5)	55 (54.5)	0.17
Positive	105 (100)	38 (36.2)	67 (63.8)	
**Cough**				
Negative	122 (100)	59 (48.4)	63 (51.6)	0.008∗
Positive	84 (100)	25 (29.8)	59 (70.2)	
**Sore throat**				
Negative	145 (100)	58 (40)	87 (60)	0.72
Positive	61 (100)	26 (42.6)	35 (57.4)	
**Inflammation of the eye**				
Negative	189 (100)	79 (41.8)	110 (58.2)	0.31
Positive	17 (100)	5 (29.4)	12 (70.6)	
**Sense of smell**				
Lost	113 (100)	46 (40.7)	67 (59.3)	0.98
Present	93 (100)	38 (40.9)	55 (59.1)	
**Sense of taste**				
Lost	134 (100)	54 (40.3)	80 (59.7)	0.84
Present	72 (100)	30 (41.7)	42 (58.3)	
**Diarrhea**				
Negative	176 (100)	68 (38.6)	108 (61.4)	0.13
Positive	30 (100)	16 (53.3)	14 (46.7)	
**Shortness of breath**				
Negative	151 (100)	66 (43.7)	85 (56.3)	0.15
Positive	55 (100)	18 (32.7)	37 (67.3)	
**Chest discomfort**				
Negative	163 (100)	71 (43.6)	92 (56.4)	0.11
Positive	43 (100)	13 (30.2)	30 (69.8)	

#Values are reported as a number (percentage) or standard deviation of the mean. ∗ Significant at the 0.05 level.

Association of Specific-IgG with clinical characteristics of the patients based on time elapsed since recovery.

In the present study, we attempted to characterize the association between Specific-IgG clinical characteristics of the patients based on time of the test in 4 different groups (2, 4, 6, 8 months after the recovery). Our data show that malaise (*P* = 0.007) in group 1, sweating (P = 0.004) in group 2, nausea (P = 0.022) in group 3, and diarrhea (P = 0.045) in group 4 had a significant relationship with the positive presence of specific COVID-19 antibodies (data not shown).

### 3.3 Association of Specific-IgG presence and COVID-19 disease severity (mild, moderate, and severe)

The rate of positive antibodies in patients with mild, moderate, and severe disease was 51.5%, 64%, and 78.9%, respectively, which showed a significant difference in the frequency distribution of IgG antibody positive cases and to the severity of the disease, according to the results of Chi-Square test (P < 0.05) (*P* = 0.042) (Table 3).

**Table 3:** Frequency distribution of positive Specific-IgG cases according to disease severity

Variables	Total	IgG		P-value
		Negative Positive		
**Severity of the disease**				
Mild	101 (100)	49 (48.5)	52 (51.5)	
Moderate	86 (100)	31 (36)	55 (64)	0.001∗
Severe	19 (100)	4 (21.1)	15 (78.9)	

#Values are reported as numbers (percentages). ∗ Significant at the 0.05 level.

### 3.4 Association of Specific-IgG presence and time elapsed since recovery

According to Table 4, the rates of positive IgG antibodies in 2, 4, 6, 8 months after the recovery were 80.8%, 69.1%, 43.2%, 41.8%, respectively. The level of IgG antibodies decreases with time after the patient recovers, and according to the Chi-Square test results, there was a significant relationship between the time elapsed after recovery and the result of the IgG test (*P* < 0.01).

**Table 4:** The relationship between the Specific-IgG test results and time elapsed since recovery

Variables	Total	IgG		P-value
		Negative Positive		
**Time elapsed since recovery**				
2 months	52 (100)	10 (19.2)	42 (80.8)	
4 months	55 (100)	17 (30.9)	38 (69.1)	
6 months	44 (100)	25 (56.8)	19 (43.2)	0.001∗
8 months	55 (100)	32 (58.2)	23 (41.8)	

#Values are reported as numbers (percentages). ∗ Significant at the 0.05 level.

### 3.5 The multiple logistic regression analysis

The multiple logistic regression model showed that the variables of age and the time elapsed after recovery had a significant relationship with the positive antibody test of recovered COVID-19 patients (P < 0.05). But other variables had no significant relationship with the result of antibody test (P > 0.05) (Table 5).

**Table 5:** The relationship between the risk factors and Specific-IgG test results by the multiple logistic regression model

Variable (base level)	AOR^#^ (%95 CI)	P
**Age**	1.06 (1.03, 1.09)	0.001^a^
**Education** (diploma and below)		0.71
Higher than diploma	1.18 (0.46, 3.01)	
**Occupation** (housekeeper)		
Employee/Retired	0.76 (0.23, 2.47)	0.65
other	1.05 (0.31, 3.58)	0.92
**Hospitalization** (No)		
Yes	2.05 (0.48, 8.65)	0.32
**Underlying disease** (No)		
Yes	3.35 (0.82, 13.70)	0.09
**Severity of the disease** (Mild)		
Moderate	0.78 (0.18, 3.25)	0.73
Severe	0.67 (0.09, 4.78)	0.69
**Time elapsed since recovery** (2 months)		
4 months	0.54 (0.20, 1.45)	0.22
6 months	0.13 (0.04, 0.38)	<0.001^a^
8 months	0.11 (0.04, 0.31)	<0.001^a^

^**#**^ Adjusted Odds Ratio.

a Statistically significant at the 0.05 level.

## 4 Discussion

The emergence and global spread of the SARS-CoV-2 virus have become a significant threat to humans. This virus's many changes and mutations cause the diversity of its epidemiological characteristics and clinical manifestations [15,16]. Therefore, it is essential to investigate different aspects of this disease. Despite numerous studies on immune responses against the SARS-CoV-2 virus, many questions have not yet been fully answered. We investigated the IgG status in recovered patients during the follow-up period. Thus, we show the association between the persistence of IgG against SARS-COV-2 among recovered COVID-19 individuals with demographic characterization, clinical symptoms, time elapse after recovery, and disease severity.

According to the results, no significant relationship was observed between gender and COVID-19-specific IgG antibody presence. Our study demonstrated there is a significant relationship between the age of patients and the presence of specific IgG antibodies at any given time of the tests. A previous study reported that age was associated with increased antibody responses against SARS-COV-2 across the serological assays [17]. Convincing evidence about the associations between IgG levels and age should be considered hypothesis-generating and tested using larger samples.

Another point to be emphasized is that we also analyzed the correlation between presence of specific antibodies in patients' sera and the underlying diseases. As shown in results, there is no statistically significant difference between this variable and IgG results. This can probably be explained by the small number of COVID-19 patients with the underlying disease in our study. It is obvious that it is essential to know for individuals with underlying disease, following the recommendations for prevention of COVID-19 disease should be done with more emphasis and they also should be carefully followed for timely diagnosis and treatment [4]. In this study, we analyzed the level of SARS-CoV-2-specific IgG antibody among 206 recovered COVID-19 subjects categorized into three groups based on disease severity. This study demonstrates that patients with severe COVID-19 disease had positive IgG antibody responses for extended periods compared to those with mild and moderate subjects. In spite of this, the multivariate logistic regression method showed that there was no significantly difference between the severity of the disease and specific IgG results. Interestingly, in a systematic review conducted by Huang et al., [2020], there was a significant relationship between the level of specific antibodies and the severity of the symptoms of COVID-19 in the patients [9]. Previous studies suggested that the clinical severity of the disease is associated with SARS-CoV-2 specific serum IgG antibodies [[18], [19], [20]].

Our results confirm previous findings that the time elapsed after disease recovery is associated with lower SARS-CoV-2-specific serum-IgG antibodies [21,22], and the duration presence of antibodies is not related to sex [23,24]. We could also observe that some patients still had detectable IgG levels after more than 240 days. In a study, Wajnberg et al. showed that IgG antibody levels against SARS-CoV-2 could be detected up to an average of 82 days in hospitalized patients [25]. In another study, Long et al. reported that 97% of 37 patients with mild COVID-19 showed a decline in IgG levels after 2 to 3 months [11].

In this study, we found that approximately 18% of patients with COVID-19 did not develop detectable anti-SARS-CoV-2 IgG in their serum. We conclude that compared with IgG-positive patients, those with IgG-negative may have a relatively mild infection of COVID-19, and the negligible effect on their immune system leads to an IgG-negative test and some increase in their lymphocytes. In addition, the differences between the results may be related partly to the sensitivity and specificity of the diagnostic kit or the target antigens used in this kit.

One of the limitations of this study is that due to our budget constraints, the present study could not include certain immunologic markers such as CD4, CD8, IFN-γ, and IL-6. Another limitation of the study is that study was performed as single center on an adult population recovered from COVID-19 in the eastern part of Iran, Thus, conducting multicenter studies in different geographic regions of the world could offer a better understanding of humeral immune responses to SARS-CoV-2 and theirs effective factors.

## 5 Conclusion

Since IgG plays crucial role in the immune response, understanding IgG status in recovered patients is essential to prevent re-infection. In the present study, the presence of COVID-19 IgG antibodies decreased significantly over time after recovery. On the other hand, the retention rate of COVID-19-specific IgG was higher inpatient with severe symptoms than the patients with moderate or mild symptoms and interestingly presence of antibodies was lower in younger population. Bearing this in mind, the implementation of health protocols and the completion of vaccination courses to prevent the risk of re-infection of COVID-19 is highly recommended.

## CRediT authorship contribution statement

**Abdol Sattar Pagheh:** Conceptualization, Methodology, Software, Data curation, Writing – original draft. **Arash Ziaee:** Data curation, Writing – original draft. **Khadijeh Abrari Romenjan:** Visualization, Investigation. **Fatemeh Rezaea:** Software, Validation. **Babak Bahman:** Visualization, Investigation. **Effat Alamzadeh:** Writing – review & editing. **Samira Elhamirad:** Software, Validation. **Masood Ziaee:** Supervision.
